# A retrospective analysis of coagulation function indicators in acute fatty liver of pregnancy

**DOI:** 10.3389/fmed.2026.1840565

**Published:** 2026-06-04

**Authors:** Panpan Zhai, Jing Hu, Xueting Ou, Liyang Zhou, Xingfei Pan

**Affiliations:** 1 Department of Infectious Diseases, Guangdong Provincial Key Laboratory of Major Obstetric Diseases, Guangdong Provincial Clinical Research Center for Obstetrics and Gynecology, The Third Affiliated Hospital, Guangzhou Medical University, Guangzhou, China; 2 Department of Obstetrics and Gynecology, Department of Obstetrics, Guangdong Provincial Key Laboratory of Major Obstetric Diseases, Guangdong Provincial Clinical Research Center for Obstetrics and Gynecology, Guangdong-Hong Kong-Macao Greater Bay Area Higher Education Joint Laboratory of Maternal-Fetal Medicine, The Third Affiliated Hospital, Guangzhou Medical University, Guangzhou, China

**Keywords:** acute fatty liver of pregnancy, adverse outcomes, coagulation function, internal validation, logistic regression, risk indicator

## Abstract

**Background:**

Acute Fatty Liver of Pregnancy (AFLP) is a rare but life-threatening obstetric emergency. Coagulation dysfunction is a core pathophysiological feature and a key risk factor for adverse events. This study retrospectively analyzed 92 AFLP patients to evaluate the association between coagulation indicators and multiple adverse outcomes, and obtain primary indicator that can predict the severity of disease.

**Methods:**

This retrospective cohort study included 92 AFLP patients treated at our hospital from 2017 to 2023. We collected clinical manifestations, laboratory data, and composite adverse outcomes including ICU admission, maternal complications. Logistic regression with variance inflation factor (VIF) diagnostics was used to analyze independent correlates of adverse outcomes. Receiver operating characteristic (ROC) analysis was performed for single indicators.

**Results:**

INR (AUC 0.837) and PT (AUC 0.837) showed the strongest individual predictive performance for composite adverse outcomes, these two indicators are the mostsensitive in predicting severity of AFLP. The VIF value of PT is greater than 10, indicating severe multicollinearity. The VIF value of INR was less than 5, and was independently associated with higher rates of MODS, DIC, postpartum hemorrhage.

**Conclusion:**

Coagulation function indicators are strongly associated with adverse clinical events in AFLP. INR provides improved risk stratification performance over other markers and may support clinical decision-making. This preliminary indicator requires external validation before routine clinical use.

## Introduction

Acute Fatty Liver of Pregnancy (AFLP) is a relatively rare disease, but can have severe adverse to mother and their fetus. AFLP often occurs in the third trimester (1, 2), and the mortality of AFLP is relatively high (3, 4). The disease can rapidly progress, to acute hepatitis, liver failure, systemic inflammatory response syndrome (SIRS) and multi-organ failure (5). Furthermore, AFLP can result in all kinds of obstetric complications, such as hemorrhage, infection, prematurity, perinatal mortality and other long-term health problems (6, 7). However, it is still a major challenge for doctors to assess the severity of AFLP, which occurs predominantly in the third trimester and poses a major challenge for early risk assessment and timely intervention (5, 8).

Coagulation dysfunction is a prominent and early feature of AFLP, driven by impaired hepatic synthetic function and systemic inflammation (9, 10). Prothrombin time (PT), prothrombin time activity percentage (PT%), international normalized ratio (INR), activated partial thromboplastin time (APTT), fibrinogen (Fbg), thrombin time (TT), and antithrombin III (AT3%) are widely used markers of coagulation and liver synthetic function (11–13). For instance, APTT and Fbg reflect the intrinsic pathway and the final common pathway of the coagulation cascade, respectively. TT and AT3% reflect the efficiency of clot formation and natural anticoagulant levels (14–16). However, the incremental value these parameters in a combined panel for risk stratification in AFLP—beyond individual markers—has not been fully quantified.

ICU admission is commonly used as a surrogate for severe disease in clinical research, but it is influenced by institutional protocols and clinician judgment (17). To improve objectivity, this study used a composite endpoint including ICU admission, maternal critical complications (MODS, DIC, coagulopathy, postpartum hemorrhage). We aimed to: (1) describe coagulation abnormalities in AFLP; (2) identify independent coagulation correlates of adverse outcomes; (3) develop and internally validate a preliminary risk indicator; and (4) clarify the clinical value of the marker for early identification of high-risk patients.

## Methods

### Study design and population

This retrospective cohort study enrolled AFLP patients treated at the Third Affiliated Hospital of Guangzhou Medical University from July 2017 to July 2023. Diagnosis was based on clinical presentation, laboratory tests, and imaging, confirmed by the modified Swansea criteria (18).

Inclusion criteria: Confirmed AFLP, Complete clinical, laboratory, and outcome data.

Exclusion criteria: Pre-existing chronic liver disease (viral hepatitis, autoimmune liver disease, alcoholic liver disease), Incomplete medical records.

### Data collection

Data of this study were systematically collected from the electronic medical records system of our hospital. Demographic information (age, gestational age at diagnosis), detailed clinical presentation (symptoms like jaundice, nausea, vomiting, and abdominal pain), laboratory results (such as levels of alanine aminotransferase, bilirubin, serum creatinine, prothrombin time, fibrinogen), liver ultrasound, and outcomes (including ICU admission rates and recovery or mortality outcomes) of all patients were included. After data collection, we analyzed the correlation between them and the severity of AFLP.

### Statistical analysis

Analyses were performed using SPSS 27.0. Continuous variables: mean ± standard deviation; compared by *t*-test or Mann–Whitney *U*-test. Categorical variables: counts and percentages; compared by χ^2^ or Fisher exact test. Multicollinearity assessment: variance inflation factor (VIF), VIF <5 was considered acceptable. Logistic regression was used to identify independent correlates of adverse outcomes. ROC analysis: AUC for single coagulation indicator. *P* < 0.05 was considered statistically significant.

## Results

### Patients' clinical characteristics

92 patients with AFLP were enrolled in the present study, Nausea and vomiting were the most prevalent symptoms, affecting 34.78% of the patients (32 cases), followed by abdominal pain in 33.7% (31 cases), and jaundice in about 25% (23 cases) (Table 1). 50% of patients had symptoms after 37 weeks of gestation. 40.22% of patients had symptoms between 34 and 36 + 6 weeks. Only 9.78% of patients had symptoms before 34 weeks of gestation.

**Table 1:** Clinical symptoms of patients with AFLP.

Clinical symptoms	Number of cases	Percentage (%)
Nausea and vomiting	32	34.78
Abdominal pain	31	33.7
Jaundice	23	25
Fatigue	5	5.43

Among the 92 pregnant women, 59.78% of them were primiparous and 40.22% were multiparous. The maternal death rate was 1.09% (1/92). 22 patients (23.9%) were complicated with multiple organ dysfunction syndrome (MODS). Fourteen patients (15.2%) were complicated with postpartum hemorrhage. Other complications are relatively rare, such as Coagulopathy (8.7%), DIC (6.5%), Hypoproteinemia (4.3%) (Table 2).

**Table 2:** General clinical characteristics of patients with AFLP.

Index	Variable	Number of cases	Percentage (%)
Gravida	Primigravida	55	59.78
	Multigravida	37	40.22
Pregnant outcome	Maternal death	1	1.09
Complications	MODS	22	23.9
	Postpartum hemorrhage	14	15.2
	Coagulopathy	8	8.7
	DIC	6	6.5
	Hypoproteinemia	4	4.3
	Hepatic encephalopathy	4	4.3
	Pancreatitis	3	3.3
	Acute renal failure	1	1.1

### Rate of ICU admission and key predictive indicators

We found that 57 patients (57/92, 61.95%) were admitted to ICU. Patients undergoing cesarean were more prone to be admitted to ICU than those who had vaginal delivery (*p* = 0.027). Gestational age was another critical factor: patients <under 37 gestational weeks (42 cases) had higher ICU admission compared to those ≥37 gestational weeks (15 cases) (*p* = 0.032). Other indicators that were statistically analyzed and found to have significant differences are MODS (*p* = 0.007), Jaundice (*p* = 0.025), Diabetes (*p* = 0.024), Respiratory Infection (*p* = 0.037), Coagulation Dysfunction (*p* = 0.034), DIC (*p* = 0.019) (Table 3).

**Table 3:** Clinical factors affecting ICU admission in pregnant patients with AFLP.

Index	Variable	Maternal outcome		
		Not ICU	ICU	*p*-value
	Total	35	57	
Delivery method	Cesarean	25	52	0.027
	Natural	10	5	
Jaundice	TRUE	4	19	0.025
	FALSE	31	38	
Fatigue	TRUE	3	2	0.365
	FALSE	32	55	
Nausea and vomiting	TRUE	9	23	0.228
	FALSE	26	34	
Abdominal pain	TRUE	12	19	1
	FALSE	23	38	
Gestational week	>Median 37.0	15	15	0.032
	≤ Median 37.0	20	42	
Diabetes	TRUE	14	23	0.024
	FALSE	21	34	
Respiratory infection	TRUE	4	15	0.037
	FALSE	31	42	
Coagulation dysfunction	TRUE	0	8	0.034
	FALSE	35	49	
DIC	TRUE	0	6	0.019
	FALSE	35	51	
Hypoproteinemia	TRUE	1	3	0.245
	FALSE	34	54	
Acute renal failure	TRUE	0	1	0.431
	FALSE	35	56	
MODS	TRUE	3	19	0.007
	FALSE	32	38	
Postpartum hemorrhage	TRUE	2	12	0.058
	FALSE	33	45	
Pancreatitis	TRUE	0	3	0.92
	FALSE	35	54	
Hepatic encephalopathy	TRUE	0	4	0.075
	FALSE	35	53	

### Logistic regression model analysis

A logistic regression model was used to evaluate the effect of various biochemical and clinical parameters on the prognosis of 92 patients with AFLP. ICU admission was defined as the key outcome measure. The international normalized ratio (INR) was positively associated with the risk of ICU admission (*B* = −329.014, *p* = 0.036). The higher prothrombin time activity percentage (PT%) was, the better the outcome of the patients was, and the lower the ICU admission was (*B* = 29.803, *p* = 0.036). However, there were not statistically significant differences in parameters such as White Blood Cell Count (WBC), Red Blood Cell Count (RBC), and liver enzymes (ALT and AST) between the two groups (Table 4).

**Table 4:** Logistic regression model analysis of predictive factors for ICU admission in patients with AFLP.

Variable	*B*	*SE*	Wald	*df*	OR (95% CI)	*p*-value
WBC	0.084	0.09	0.874	1	1.088 (0.912–1.298)	0.35
Neu	−0.061	0.068	0.792	1	0.941 (0.823–1.076)	0.374
RBC	−0.991	0.917	1.168	1	0.371 (0.062–2.240)	0.28
Hb	0.062	0.036	2.861	1	1.064 (0.990–1.142)	0.091
PLT	−0.006	0.01	0.358	1	0.994 (0.975–1.014)	0.55
PT%	29.803	14.247	4.376	1	8.771E + 12 (6.547–1.175E + 25)	0.036
PT	−0.116	0.061	3.682	1	0.890 (0.791–1.002)	0.05
INR	−329.014	156.878	4.398	1	0.000 (0.000–0.000)	0.036
APTT	0.069	0.057	1.447	1	1.071 (0.958–1.198)	0.229
Fbg	1.84	1.272	2.092	1	0.159 (0.013–1.922)	0.148
TT	−0.121	0.062	3.856	1	0.886 (0.784–1.000)	0.05
AT3%	0.092	0.045	4.185	1	1.096 (1.004–1.197)	0.041
DD	0	0	1.963	1	1.000 (1.000–1.000)	0.161
ALT	−0.004	0.004	1.179	1	0.996 (0.989–1.003)	0.278
AST	0.006	0.003	3.514	1	1.006 (1.000–1.013)	0.061
Tbil	0.012	0.01	1.319	1	1.012 (0.992–1.032)	0.251
Dbil	0.039	0.021	3.351	1	1.040 (0.997–1.084)	0.067
ALB	−0.024	0.026	0.84	1	0.976 (0.928–1.027)	0.359
Urea	−0.066	0.265	0.062	1	0.936 (0.557–1.573)	0.803
Cr	0	0.011	0	1	1.000 (0.978–1.022)	0.989
Blood ammonia	0.003	0.033	0.01	1	1.003 (0.940–1.071)	0.919
BNP	−0.012	0.015	0.611	1	0.989 (0.960–1.018)	0.434
TBA	0.001	0.015	0.005	1	1.001 (0.971–1.032)	0.946
Urinaryprotein	0.221	0.954	0.054	1	1.247 (0.192–8.085)	0.817
Urinarybilirubin	−0.091	1.332	0.005	1	0.913 (0.067–12.412)	0.945
liverultrasound	−0.547	0.89	0.378	1	0.579 (0.101–3.310)	0.539

### Risk indicator analysis

**Figure 1:**
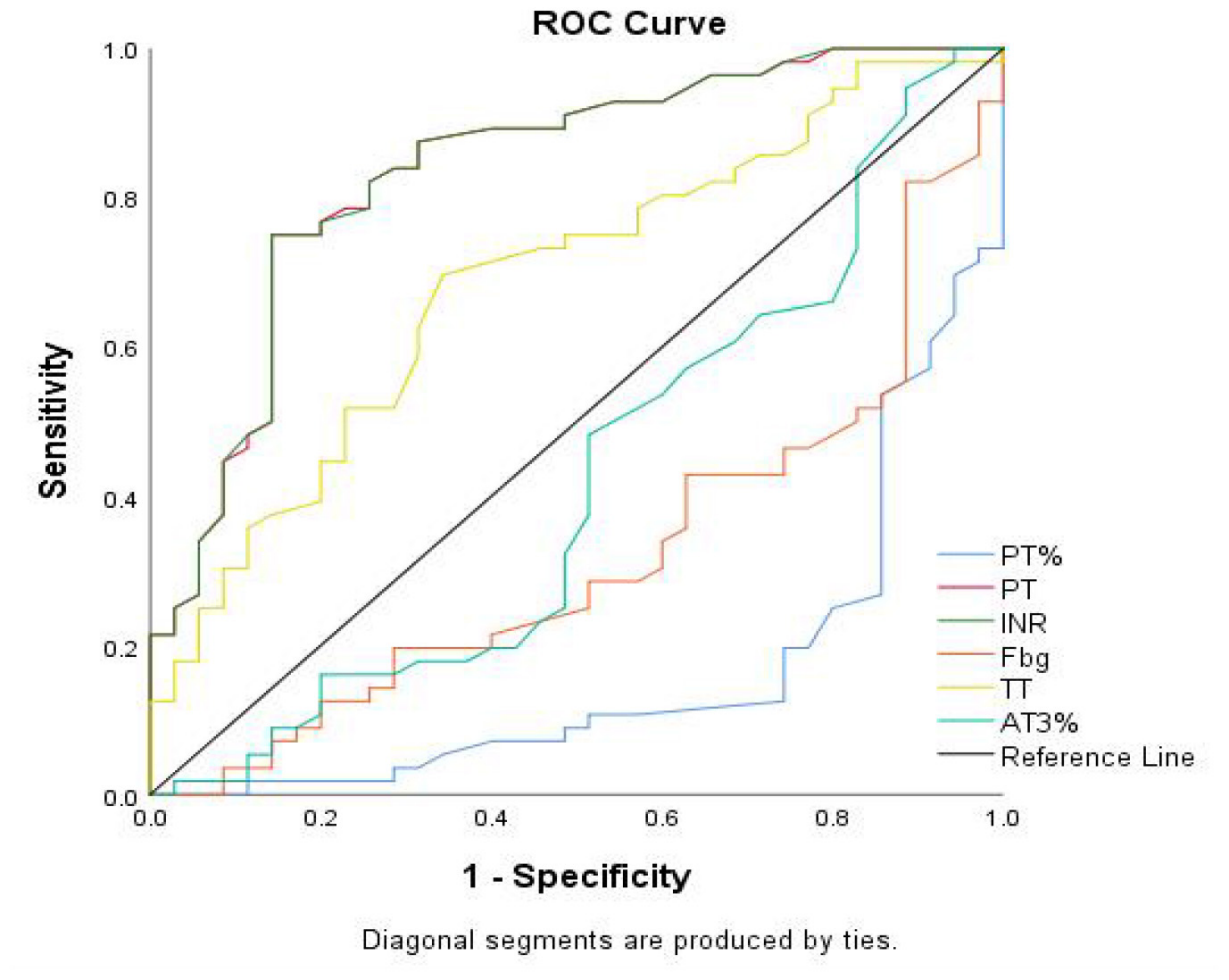
ROC curve analysis of predictive indicators for the severity of AFLP.

In Table 5 and Figure 1, among the analyzed coagulation function indicators, the AUC curves of INR and PT have high sensitivity, which can alert doctors in advance to the severity of the patients. After VIF verification, it was found that PT has a VIF > 10, while INR has a VIF <5. Through comprehensive analysis and comparison, INR seems to be the best high-sensitivity indicator for assessing the severity of AFLP in coagulation function.

**Table 5:** Predictive performance of AFLP prognostic model.

Variable	AUC	SE	95% CI	*p*-value
PT%	0.161	0.044	0.075–0.247	<0.001
PT	0.837	0.044	0.751–0.924	<0.001
INR	0.837	0.044	0.751–0.924	<0.001
Fbg	0.320	0.057	0.208–0.432	0.004
TT	0.688	0.056	0.578–0.799	0.056
AT3%	0.420	0.064	0.294–0.546	0.202

### Clinical factors affected by INR

In the above logistic regression analysis, whether the patient was admitted to the ICU was used as anindicator for assessing the severity of the condition. To avoid the influence of the subjective factor of ICU admission determined by doctors, and to further verify the predictive value of the INR indicator for the severity of AFLP, INR was re-validated internally through pregnancy complications, postpartum hemorrhage, MODS, and other factors. The results showed that INR independently associated with higher rates of MODS, DIC and postpartum hemorrhage (Table 6).

**Table 6:** Clinical factors affected by INR in pregnant patients with AFLP.

Index	Variable	INR		
		≤ 1.5	>1.5	*p*-value
	Total	56	36	–
Jaundice	TRUE	9	14	0.014
	FALSE	47	22	
Fatigue	TRUE	1	4	0.054
	FALSE	55	32	
Nausea and vomiting	TRUE	19	13	0.830
	FALSE	37	23	
Abdominal pain	TRUE	22	9	0.021
	FALSE	34	27	
Diabetes	TRUE	23	14	0.835
	FALSE	33	22	
Respiratory infection	TRUE	6	13	0.003
	FALSE	50	23	
DIC	TRUE	0	6	0.002
	FALSE	56	30	
Hypoproteinemia	TRUE	3	1	0.554
	FALSE	53	35	
Acute renal failure	TRUE	1	0	0.462
	FALSE	55	36	
MODS	TRUE	3	19	<0.001
	FALSE	53	17	
Postpartum hemorrhage	TRUE	2	12	<0.001
	FALSE	54	24	
Pancreatitis	TRUE	1	2	0.320
	FALSE	55	34	
Hepatic encephalopathy	TRUE	0	4	0.011
	FALSE	56	32	

## Discussion

Previous studies have demonstrated that Acute Fatty Liver of Pregnancy (AFLP) predominantly occurs in the late trimester of pregnancy, typically after 30 gestational weeks (13, 19, 20). Aligning with prior research, our findings confirmed that the majority of AFLP cases mainly occurred in the late trimester of pregnancy, particularly after 37 gestational weeks. Physiological and metabolic changes during the third trimester of pregnancy might increase the morbidity of AFLP. For example, the increase of maternal and fetal metabolic demand could aggravate the burden of liver function (15). Moreover, hormonal fluctuations in late pregnancy might affect liver function, potentially triggering the occurrence of AFLP (16).

Symptoms of AFLP, particularly in its initial stage, are atypical and may be easily overlooked. Typical clinical signs include malaise, anorexia, nausea, vomiting, abdominal pain, jaundice, ascites, hypertension, and distension (9, 21, 22). Our results revealed that anorexia, nausea, vomiting, and abdominal pain were prevalent prodromal symptoms, while progressive jaundice and ascites were common clinical manifestations. In the present study, nausea and vomiting were the most frequent symptoms, affecting 34.78% of patients (32/92), followed by abdominal pain in 33.7% of patients (31/92), and jaundice in about 25% of patients (23/92). Only 5.43% of patients showed fatigue (5/92). Our results above implied that we should be alert to patients with nausea, abdominal and jaundice during the late trimester of pregnancy because they could have AFLP. Moreover, the occurrence of jaundice was significantly correlated with the severity of AFLP. Our results were similar to previous studies which reported that the increase of jaundice was thought not only as a direct indicator of liver dysfunction but also as a marker of disease severity (23, 24). Additionally, AFLP was often complicated with multi-organs dysfunction, and was necessarily treated as soon as possible.

Diagnostically, AFLP is primarily identified by using the Swansea criteria (13, 25), known for the high accuracy and ability to timely facilitate interventions. Although liver biopsy remains the definitive standard for diagnosing AFLP, it is infrequently performed due to the pronounced coagulopathy in these patients and the procedure's invasive nature, which could result in substantial risks (26, 27). Furthermore biopsy is rarely done for cases with unclear etiologies or postpartum. Taken together, it is very important to find laboratory diagnostic markers, especially infection markers and liver failure indicators. These markers are crucial for non-invasive diagnostics and provide essential insights into the disease progression (28–30). For instance, common infection markers like C-reactive protein (CRP) and white blood cell counts could reflect the body's inflammatory state, while liver failure indices such as transaminases (ALT and AST), bilirubin, and the International Normalized Ratio (INR) could indicate liver damage and coagulation abnormality. In the present study, there was a significant difference in INR or PT% (mean INR was 1.6 ± 0.8, 73.63% abnormal; mean PT was 17.1 ± 8.4, 73.91% abnormal) in patients with AFLP, respectively. Our results further demonstrated that these parameters played important roles in the early diagnosis of AFLP and reflecting the severity of AFLP. Furthermore, we specifically paid attention to the association between coagulation function indicators and the incidence of ICU admission. We found that compared to that of patients who were not transferred to ICU, patients transferred to ICU had the decrease of PT%, Fbg, the increase of INR, TT, PT, APTT. Taken together, we think that abnormal coagulation indicators could be correlated with the severity of AFLP.

Given the rapid progression and high mortality of AFLP (31), it is very important to develop clinical predictive indicators for AFLP (32). The use of coagulation function markers could effectively predict the severity of disease and the prognosis of patients with AFLP. In this study, we found that high INR value was closely related to the poor prognosis of AFLP.

However, our study also had some limitations. Firstly, the sample size was relatively small. Secondly, the present study was a retrospective study. More cases with AFLP should be enrolled and perspective researches should be done in order to demonstrate our results in the future. Additionally, researches should focus on improving early diagnostic methods and developing personalized treatment strategies to reduce AFLP complications and enhance maternal survival rates.

## Conclusions

In sum, this study conducted a retrospective analysis of clinical data collected from 92 patients with AFLP. We explored the relationship between coagulation function markers and liver failure indices with disease severity, and found that INR value was closely related to the prognosis of AFLP. This preliminary indicator requires external validation before routine clinical use.

## Data Availability

The raw data supporting the conclusions of this article will be made available by the authors, without undue reservation.
